# Uma companhia inesperada? O cão de serviço e a expressão de emoções no espaço hospitalar: uma perspectiva etnográfica

**DOI:** 10.1590/0102-311XPT024925

**Published:** 2025-11-07

**Authors:** Roberta Falcão Tanabe, Anita Silva Paez, Paula de Araújo Picorelli, Vanessa Christina Breia, Martha Cristina Nunes Moreira

**Affiliations:** 1 Instituto Nacional de Saúde da Mulher, da Criança e do Adolescente Fernandes Figueira, Fundação Oswaldo Cruz, Rio de Janeiro, Brasil.; 2 Faculdade de Formação de Professores, Universidade do Estado do Rio de Janeiro, Rio de Janeiro, Brasil.

**Keywords:** Intervenção Assistida por Animais, Etnografia, Trabalhadores da Saúde, Hospitais Pediátricos, Promoção da Saúde, Animal Assisted Interventions, Ethnography, Healthcare Workers, Pediatric Hospitals, Health Promotion, Intervenciones Asistidas con Animales, Etnografía, Trabajadores de la Salud, Hospitales Pediátricos, Promoción de la Salud

## Abstract

A relação humano-animal no espaço de cuidado hospitalar foi investigada a partir de uma pesquisa etnográfica na observação de um programa de apoio assistido por animais (PAAA) em uma instituição terciária do Sistema Único de Saúde no Rio de Janeiro, Brasil. O artigo enfoca os efeitos de interação comunicativa e os agenciamentos que a visita regular de um único cão de serviço parece catalisar entre os membros da equipe de trabalhadores. Tomando como suportes analíticos a Teoria Ator-Rede e a noção de “espécie companheira” de Donna Haraway exploramos a reconfiguração das redes de sociabilidade na integração de uma alteridade não-humana como colaboradora das atividades de promoção de saúde no cenário hospitalar. Ao operar por lógicas não norteadas pelo poder e racionalidade, seu papel político é destacado nos diferentes agenciamentos produzidos na dinâmica relacional e na economia dos afetos. Os novos arranjos instituídos pelo cão de serviço delineiam a dimensão micropolítica das emoções na reorganização das relações interpessoais e interespécies no espaço de cuidado hospitalar. A pesquisa evidencia que o PAAA em um hospital pode operar como uma tecnologia social inovadora em saúde, capaz de reconfigurar as redes de cuidado e as dinâmicas de interação.

## Abstract (en)

The human-animal relationship in the hospital care setting was investigated based on an ethnographic study of an animal-assisted support program at a tertiary institution within the Brazilian Unified National Health System in Rio de Janeiro, Brazil. This study examines the effects of communicative interactions and the agency that regular visits by a single service dog seem to catalyze among staff members. Using the Actor-Network Theory and Donna Haraway’s concept of “companion species” as analytical frameworks, we explore the reconfiguration of social networks in the integration of a non-human other as a collaborator in health promotion activities within hospital settings. By operating according to logics not driven by power or rationality, its political role is highlighted in the various agencies produced within relational dynamics and the economy of affects. The new arrangements introduced by the service dog outline the micropolitical dimension of emotions in the reorganization of interpersonal and interspecies relationships within hospital settings. This study shows that animal-assisted support progra in hospitals can function as an innovative social technology in health, capable of reconfiguring care networks and interaction dynamics.

## Abstract (es)

La relación humano-animal en el ámbito de atención hospitalaria se evaluó a partir de una investigación etnográfica realizada mediante la observación de un programa de apoyo asistido por animales en una institución terciaria del Sistema Único de Salud en Rio de Janeiro, Brasil. Este artículo se centra en los efectos de la interacción comunicativa y de la expresión que la visita regular de un solo perro de servicio parece catalizar entre los miembros del equipo de trabajadores. A partir de los aportes de la Teoría del Actor-Red y de la noción de “especie compañera” de Donna Haraway, este estudio explora la reconfiguración de las redes de sociabilidad en la integración de una alteridad no humana como colaboradora de las actividades de promoción sanitaria en el ámbito hospitalario. Al operar lógicas no guiadas por el poder y la racionalidad, su papel político se destaca en las diferentes agencias producidas en las dinámicas relacionales y en la economía de los afectos. Los nuevos arreglos que se generan con el perro de servicio describen la dimensión micropolítica de las emociones en la reorganización de las relaciones interpersonales e interespecies en la atención hospitalaria. Este estudio señala que el programa de apoyo asistido por animales en un hospital puede operar como una tecnología social innovadora en salud, capaz de reconfigurar las redes de atención y las dinámicas de interacción.

“*Saber que um cachorro entende linguagens que seres humanos já nem sabem mais*” (trecho da música *Eu na Máquina Vou*, autoria de Silvério Pessoa, Alceu Valença e Zé Vicente da Paraíba).

## Introdução

A rede de cuidado tecida em cenas de hospitalização prolongada de crianças e adolescentes vivendo com condições crônicas, complexas e raras de saúde constitui-se em uma intrincada trama, composta por diversos atores humanos e não-humanos, numa tessitura de negociações nas quais emoções e acordos se atualizam dinamicamente ^1^. Para esse grupo de crianças e adolescentes, o hospital é muitas vezes um local de permanência prolongada, o que exige estratégias de atenção à saúde nas quais o cotidiano se configure em um espaço em que a infância e a vida possam ser valorizadas ^2^. Retomamos aqui as bases da Teoria Ator-Rede (TAR) de Bruno Latour ^3^, destacando que a cada novo encontro estas redes se reconfiguram de acordo com a dinâmica das interações entre os atores envolvidos e o fluxo de emoções, forças, motivações e interesses em questão.

Tratamos neste artigo dos efeitos da interação de trabalhadores de saúde com uma cadela de serviço em um hospital público terciário. Recorremos à Haraway ^4^ e à noção de espécie companheira para pensar a alteridade neste contexto. Agregar novos atores, como os animais, na composição de uma concepção ampliada do que seja o outro nos faz deslocar o conceito de social como “*espaço exclusivo dos fazeres humanos*” ^4^ (p. 135). Nosso ponto de inflexão aqui se sustenta na experiência em curso, há mais de um ano, de um programa de apoio assistido por animais (PAAA), que tem a participação de uma cadela Golden Retriever na visita semanal a um instituto federal de saúde situado no Rio de Janeiro, Brasil.

Os serviços assistidos por animais (SAA) têm despertado interesse crescente nas últimas décadas. Esse termo guarda-chuva abarca um conjunto de três modalidades: educação assistida por animais; tratamento assistido por animais; e programas de apoio assistido por animais ^5^. Publicações nacionais e internacionais enumeram os múltiplos benefícios fisiológicos, socioemocionais, motores, cognitivos, psicológicos e mentais do contato e convívio com animais em diferentes grupos estudados ^6^ ^,^ ^7^ ^,^ ^8^ ^,^ ^9^. Na atenção pediátrica, a presença dos animais parece produzir efeitos significativos como adjuvante ao tratamento clínico de pacientes hospitalizados, por exemplo, na redução da sensação de dor ^10^ ^,^ ^11^.

Quando a iniciativa em questão foi concebida, a motivação era poder oferecer a oportunidade de contato com um cão às crianças e adolescentes que, pela natureza complexa de sua condição clínica, estando em internações prolongadas e/ou recorrentes, não têm a chance de interagir fisicamente com qualquer animal. A suposição inicial era de que o cachorro fosse gerar um interesse e desejo natural por parte do público infantil por seu caráter extraordinário neste cenário específico. A experiência em curso, entretanto, tem nos revelado que a interação humano-animal também tem efeitos bastante significativos entre os trabalhadores do hospital, operando como possível suporte para a expressão de emoções e questões relacionadas ao que não se consegue dizer no cotidiano.

Motivadas por este achado que nos surpreendeu, a interação entre a cadela, como um ator inesperado, e a equipe ocupa aqui o centro de nossa análise. De que forma a presença da cadela de serviço remodela as redes que conectam humanos e não-humanos neste cenário? Enfocaremos a discussão na função dialógica que o animal parece exercer, catalisando emoções, servindo de apoio para a expressão destas e também como suporte à interlocução entre pares. A TAR será nosso referencial analítico.

Assim, o artigo se propõe a investigar os efeitos das interações humanas e não-humanas e os agenciamentos produzidos pela presença animal, por meio da exploração da experiência de visitação de uma cadela de serviço em uma instituição terciária do Sistema Único de Saúde (SUS) brasileiro, sustentando as possibilidades desta iniciativa como uma tecnologia inovadora na promoção de saúde para pacientes, familiares e trabalhadores.

## Metodologia

A base desta pesquisa é a Etnografia ^12^. A imersão no cotidiano e a perspectiva simbólica e interacional, reflexiva e crítica, permitem a produção de um olhar investigativo sobre o comum e ordinário das coisas. O material empírico emerge do acompanhamento planejado e técnico, apoiado por uma equipe de saúde responsável pelas atividades de caráter lúdico e terapêutico para crianças e adolescentes que vivem longas internações por conta de condições de saúde crônicas, raras e complexas ^13^. O trabalho com a visitação animal transcorre há 17 meses, após cuidadoso planejamento e preparação criteriosa do cão de serviço para o contexto e público a ser atendido.

A tutora da cadela de serviço é servidora do hospital e pesquisadora parceira do grupo. Tendo tido contato com o PAAA de outra instituição, propôs-se a investir na avaliação e preparação do seu animal para o trabalho de visitas a crianças e adolescentes. Após ser tecnicamente avaliada por veterinário e por profissional habilitado em SAA, a cadela iniciou, aos três anos de idade, seu treinamento específico com encontros semanais ao longo de seis meses. “*O treinamento é um trabalho essencialmente relacional* (...) *e envolve um processo de familiarização humano-cão que cria, além de reconhecimento de liderança, uma relação afetiva recíproca*” ^14^ (p. 42). Na delicadeza das interações humano-animal, busca-se uma sintonia fina e o desenvolvimento mútuo de habilidades associadas à leitura refinada de gestos e intenções ^15^. Nessa fase a cadela foi sendo exposta aos objetos, dispositivos médicos, público e circunstâncias que iria encontrar no ambiente hospitalar, por meio de treinamento com reforço positivo para as respostas e comportamentos que deveria adotar.

A cadela de serviço atua exclusivamente no hospital investigado e foi treinada considerando as particularidades e especificidades do público e contexto do local. Antes de começar a visitar efetivamente as crianças internadas, passou algumas semanas vindo regularmente ao espaço hospitalar para se familiarizar com o ambiente, sempre na companhia de seu treinador e condutor, com quem já possuía vínculo afetivo e de confiança. Esse profissional contava com experiência prévia na condução de outros animais em PAAA no contexto de crianças em internação hospitalar. Com intenção de aproximação maior com o animal, a pesquisadora coordenadora da visita interage com a tutora e a cadela fora do hospital, compartilhando momentos de lazer em encontros nos finais de semana.

O deslocamento da cadela de sua casa para o trabalho ocorre uma vez por semana, sempre em companhia de seu treinador em veículo institucional e dura cerca de cinco minutos. A viagem de carro e as visitas ao hospital revelaram-se, de acordo com observação da tutora e do treinador, momentos de prazer aguardados com animação pelo animal.

O fato de este programa estar vinculado a um núcleo institucional com foco na promoção da saúde, que atua há três décadas no âmbito da assistência, ensino e pesquisa, permite um alcance de profissionalização e compromisso com a produção de conhecimento e novas tecnologias em saúde. Entendemos que, por desenvolver atividades lúdicas de base interdisciplinar, este núcleo operou como um lugar seguro para a iniciativa, oferecendo um olhar zeloso do bem-estar de humanos e do animal que chegava ao ambiente como novidade. A rotina estabelecida simultaneamente ao PAAA preservou a atuação da assistência já anteriormente desenvolvida pelo grupo e integrou a esta outros participantes da equipe de pesquisa. A etnografia foi conduzida, portanto, de forma programada e negociada, integrada aos atendimentos deste núcleo já desenvolvidos rotineiramente no setor de internação do hospital.

Duas pesquisadoras atuaram de forma transversal em todos os momentos da visita. Uma delas era responsável pela organização da atividade dando suporte técnico e operacional, enquanto a outra se ocupava exclusivamente da observação e da tomada de notas para a elaboração do diário de campo. Essa multiplicidade de olhares resultou em uma percepção caleidoscópica da cena abarcando a observação dos momentos antes da chegada do cão, durante sua visita e após sua saída.

A visita acontece semanalmente, conforme dito anteriormente, e para apenas um setor por vez, com duração variando de 40 a 60 minutos, em um sistema de rodízio que contempla todas as unidades clínicas da internação pediátrica. A cada dia, desde o momento da sua chegada e de seu condutor, forma-se uma pequena aglomeração de trabalhadores buscando interagir com a cadela e tirar fotos. A situação, vista inicialmente pela equipe de pesquisa como um evento desfavorável por atrasar o início da atividade e desgastar o cachorro, pôde ser finalmente assumida como parte do trabalho. A partir de então, a atividade passou a ser organizada de forma a acolher o fluxo espontâneo de um público crescente de crianças e adultos (familiares e profissionais do hospital), que se aproximam e buscam a interação com o cão nos deslocamentos que este faz desde sua chegada até a saída do hospital. As cenas de interação observadas se desdobraram, portanto, em diferentes espaços, para além do que seria a programação de visitação de cada dia. Após a conclusão de cada visita, todo o grupo de pesquisa participou de reuniões em que eram compartilhadas as impressões e gravado um arquivo de áudio com contribuições individuais na composição de uma observação coletiva das interações daquele dia. Este registro em áudio, as fotografias e vídeos dos momentos de interação com o animal e as notas escritas no caderno de campo constituíram o conjunto material que consubstanciou a produção do diário de campo.

A análise do acervo reunido se deu a partir da perspectiva da TAR ^3^. O conceito de performance é central para estas lentes teórico-interpretativas. Este conceito, diferentemente da ideia de papel social, encara a interação como transformadora dos supostos lugares e funções esperadas. Nesse sentido, é importante considerar a cadela de serviço, nesta etnografia, como um actante que introduz novas conexões e associações a partir das interações interespécies. Ao participar do espaço hospitalar, os significados a ela atribuídos diferem daqueles que circulam quando está na sua casa ou na rua. Outras possibilidades de contato e uma sociabilidade ampliada se inscrevem quando atores humanos e não humanos interagem na cena de cuidado. Nesse deslocamento, há uma reformulação das tramas sociais operantes e dos atores legitimamente reconhecidos como passíveis de ali interagirem. Na suspensão dos enquadres pré-determinados, a presença da cadela de serviço pode ensejar novas práticas locais a partir da mobilização de outros referenciais e agenciamentos.

Como neste artigo escolhemos tratar dos efeitos de interação comunicativa que a presença da cadela de serviço parece catalisar entre os membros da equipe de trabalhadores, organizamos o material etnográfico em dois eixos a partir da localização destas cenas específicas. O primeiro eixo trata da mediação da cadela na expressão de emoções no espaço hospitalar. No segundo eixo, exploraremos as enunciações dirigidas ao animal, ou melhor dizendo, as enunciações que se apoiam na sua presença para atingir um outro ou outros interlocutores, além de destacarmos a produção de uma nova dinâmica interpessoal entre os trabalhadores.

## Resultados e discussão

### Cão como mediador da expressão de emoções no espaço de cuidado hospitalar

O cão foi o primeiro animal a ser domesticado ^16^. Com um histórico longo de convivência com humanos, desenvolveu habilidades que o qualificam como companheiro por excelência. Sua boa disposição para a interação social se configura como um potencial elemento mobilizador de interesses e afeto. Dessa forma, ocupa um espaço privilegiado nas relações com humanos, tanto por sua aparência, que evoca ternura e acolhimento, quanto por não demandar interações verbais. No ambiente de internação, onde os profissionais vivem um cotidiano dominado pela tensão, pela necessidade de decisões rápidas e de controle emocional, a chegada do animal parece estabelecer um espaço no qual a leveza e a espontaneidade podem ser experimentadas. O contato físico, buscado ansiosamente por muitos nesses encontros, parece um continente para as frustrações e angústias vividas no ambiente de trabalho.

A natureza do trabalho em uma instituição hospitalar, notadamente em setores de internação de crianças e adolescentes com doenças crônicas e graves, exige um manejo especial das emoções. Mansano ^17^ destaca a dimensão imaterial deste trabalho árduo e não contabilizável. O autocontrole emotivo é uma exigência sobre o sujeito nas sociedades ocidentais modernas, o que resulta na moderação dos afetos ^18^. Os conceitos de emoção, nesta perspectiva, devem ser vistos como elementos de práticas ideológicas locais: as emoções são um idioma que define e negocia as relações sociais entre uma pessoa e as outras. Cada cultura tem suas “regras de exibição”. Cada indivíduo, com base na sua experiência e posição social, aprende como deve expressar suas emoções e que emoções devem ser expressas ^18^. Há um interjogo entre o caráter social e culturalmente construído das emoções e a apropriação individual de valores e sentidos compartilhados, que desenham a tonalidade singular dos comportamentos num contexto particular ^19^. Dessa maneira, existem expectativas implícitas do grupo de pertença que prescrevem os repertórios adequados, a modulação da intensidade, da duração, da localização e a normatividade dos comportamentos. A relação com os pares serve, portanto, como baliza para o ajuste da expressão das emoções.

Diante da cadela de serviço, parece que os limites do permitido, em termos de expressão emocional, são ampliados.

“*Não chora não, amiga*”.

“*Eu vou chorar! Eu sou apaixonada por essa cachorra. Eu viro criança de novo*” (conversa entre enfermeiras da unidade intermediária).

“*Nossa, é hoje que perco meu emprego! Se acontecer, vai ter sido por uma boa causa. Estou muito emocionada, enfim consegui me encontrar com ela!*” (ascensorista responsável pelo elevador de acesso aos setores clínicos do prédio principal, ao abandonar seu posto e se ajoelhar no chão do hall para afagar e tirar fotos com o cão de serviço e assim perder o controle do elevador, que seguiu sem ela).

O entendimento do choro como uma expressão infantilizada e como um elemento que atrapalha ou embaça as relações de trabalho é algo digno de nota. Nestes lampejos de expressão, torna-se reconhecível a existência tácita de uma etiqueta, de uma forma esperada de como se comportar nas cenas públicas. Nas interações sociais, esse trabalho emocional se refere ao exercício de confrontar um padrão de referência, um sentimento ideal, adequando as expectativas ao processo emocional interno e sua expressão ^20^. Ao revelar-se como inesperada, a emoção precisa ser justificada, negociada ou nomeada em voz alta, como nos excertos acima.

Nos encontros com o animal, observamos a necessidade não apenas de aproximação e do registro fotográfico do momento, mas também de uma certa intimidade materializada em carinhos, abraços e beijos. Essa disponibilidade para um contato físico afetuoso sublinha um espaço de trocas onde as pessoas se autorizam a ser mais espontâneas e livres.

Haraway, em seu livro *Manifesto das Espécies Companheiras: Cães, Pessoas e Alteridade Significativa* ^4^, ilumina as relações de parceria estabelecida com os demais seres que convivem com os humanos, identificando tal presença como uma alteridade significativa. Essa alteridade não-humana produz uma sociabilidade cujos parâmetros e lógica de sustentação são distintas das que organizam os relacionamentos inter-humanos.

Ao assumir sua tarefa de visitação regular ao espaço hospitalar, nossa colaboradora canina não é apenas um animal no apoio a um serviço no âmbito da saúde. Ela passa a representar, simbólica e afetivamente, todos os animais de estimação que habitam a história de cada um, materializando cada um deles. À cadela de serviço são canalizadas e transferidas emoções e afetos que dizem respeito a muitos outros animais, inclusive de outras espécies, que são ou foram amados. Observamos que a manifestação espontânea do animal parece suscitar uma resposta menos contida por parte das pessoas. Nesses encontros com os trabalhadores, algumas emoções puderam ser liberadas e não foi infrequente a presença de lágrimas.

Nas manifestações das emoções, o significado das lágrimas depende do simbolismo de uma dada sociedade, não sendo, portanto, unívoco ^19^. Nos encontros com o cão de serviço, as lágrimas traduziram sentimentos diversos. Algumas vezes, a presença da cadela evocava a lembrança emocionada do próprio animal de estimação do momento presente ou do passado. A saudade daqueles podia ser extravasada na interação com ela. O animal passou a funcionar como um elo entre passado e presente e com as experiências vividas fora do ambiente hospitalar. Em outras ocasiões, as lágrimas representavam a expressão de alívio por estar na presença de um ser com quem se poderia dividir alguns momentos de paz, alguém que não demandaria explicações e aceitaria tranquilamente seu silêncio. Em outras, ainda, as lágrimas ficaram indecifráveis. Assim como nossa colaboradora canina, nós pesquisadoras em campo apenas presenciamos em silêncio a cena, preservando a delicadeza e a intimidade daquele momento. Conforme a TAR ^3^, podemos considerar que a cadela ocupou diferentes papéis e funções em cada interação, de acordo com os atores, as circunstâncias, o momento individual e os locais em que aconteciam os encontros.

Um dos pré-requisitos para a seleção de um cão de serviço para atuar no PAAA, além da sua docilidade, previsibilidade e obediência, é que não vocalize nas interações ^21^. Essa presença silenciosa e afável significa para muitos um acolhimento sem julgamento. Algumas vezes, profissionais se aproximaram dela para dizer segredos ao pé do ouvido. Uma interação privada como quem se dirige a um confessionário, no qual poderiam expressar genuinamente seus sentimentos sem temerem avaliações preconceituosas ou represálias. O cão assumia um papel apaziguador, um receptor confiável a quem podia ser compartilhada uma intimidade talvez inconfessável para outros humanos. O que ali fosse dito não corria o risco de ser transmitido para além daquele espaço seguro. Nessas ocasiões, permanecia sentada e quieta junto a pessoa, numa postura receptiva.

Os cães são capazes de ler as emoções humanas e ajustar-se a elas de uma forma empática ^22^. Esse tipo de resposta social se verifica tanto nos contextos privados quanto nos profissionais ^22^. Nesse sentido, conseguem mobilizar a afeição e estima nos humanos, o que é útil e valioso na atuação nos serviços assistidos por animais.

O afrouxamento das regras implícitas na expressão de emoções é acompanhado por uma relativa ruptura de regras sociais como a hierarquia. Em um espaço regido pela racionalidade, a chegada de um ser mobilizado pela sensibilidade e espontaneidade, uma presença de caráter extraordinário neste cenário específico, traz mudanças paradigmáticas no campo das interações. O cão examina e registra informações a partir de uma sensorialidade ampliada, captando aferências sobre o espaço, o campo interacional e as próprias pessoas que são extraordinárias e por vezes inacessíveis aos sentidos humanos. Ter a companhia de um parceiro de trabalho com tais atributos institui novos arranjos para a natureza dos encontros e das trocas estabelecidas. Pode-se dizer que a inclusão de um ator não-humano na cena hospitalar aciona outros interesses e catalisa encontros a partir de convergências inesperadas.

O interesse olfativo atrai o cão para determinados humanos buscando, nos corpos e vestes de crianças e adultos, uma impregnação odorífera que deixa rastros detectáveis apenas para ele. Esse comportamento resultava, por parte dos humanos, numa evocação narrativa da história com os próprios animais de estimação iniciada com o seguinte apontamento: “ela deve estar sentindo o cheiro do meu bichinho”. A cadela de serviço se demorava nessa investigação cheirando algumas pessoas e objetos que revelavam remotamente a presença de seus pares, ou talvez fosse também instigada por outros odores, que na percepção humana seriam desagradáveis ou despertariam repulsa.

Este tipo de aproximação não aconteceria se estivéssemos considerando apenas o contato inter-humano e nem é tomado como constrangedor ou inconveniente quando parte do animal. Pelo contrário, passa a ser considerado como motivo de orgulho para aqueles “eleitos” por sua percepção particular. A partir desta busca espontânea por cheiros e pessoas, a cadela de serviço promove uma nova categoria de indivíduos a quem atribui uma diferenciação privilegiada, um *status* especial. Essa classificação obedece a outras lógicas não norteadas pela hierarquia, poder e racionalidade. Nesse sentido, instaura rupturas nas normas que habitualmente dirigem as interações no espaço hospitalar.

“*Viu, é só comigo! Tá sentindo o cheiro dos meus três vira-latas.* (...) *Isso pra mim é motivo de orgulho!*” (funcionário falando para o chefe sobre a atenção privilegiada que recebeu).

“*Ela veio me ver! Ela abanou o rabo para mim!*” (funcionário do arquivo).

Um processo de interação evoca simbolismos e expectativas, assim como referências para negociar significados. A identificação de cheiros por parte do cachorro, por exemplo, remete a uma presença ausente na cena. Um “gesto” do cachorro ganha significados de distinção e reconhecimento.

Ainda que houvesse um roteiro de visita programado para cada dia, acompanhar os desdobramentos da presença do animal exigiu das pesquisadoras mais flexibilidade no planejamento e condução das visitas. Na organização do trabalho, consideramos a natureza senciente e sociável da parceira canina, admitindo que seu interesse e desejo não estavam sob a ingerência da nossa vontade. Buscou-se atender, respeitar e valorizar o caráter espontâneo e inesperado dos encontros, incluindo atenção e apoio às escolhas do cão, tanto no que dizia respeito às pessoas a quem buscava e oferecia uma atenção diferenciada, quanto à duração dos encontros com cada um. A marca distintiva de nossa colaboradora canina é seu baixo nível de energia (tendo preferência por atividades tranquilas) e docilidade, mas também sua assertividade em anunciar seus limites e apontar os seus desejos, sempre de maneira delicada.

Latour ^3^ destaca a agência dos atores não-humanos nas relações de sociabilidade, sugerindo que sigamos suas trilhas para conhecer e compreender os efeitos e sentidos produzidos a partir da sua participação nos diferentes contextos. Foi a partir desta perspectiva que assumimos uma atenção ampla para o contexto interativo, assim como para os detalhes e singularidades dos encontros.

### Cão como suporte da expressão de mal-estar e modulação das relações de trabalho

McNicholas & Collis ^23^ destacam o efeito catalítico dos cães nas interações sociais humanas e o aumento da sensação de bem-estar. Kotrschal ^22^ afirma que, além da facilitação dos contatos sociais, a presença de um cão amigável pode ter forte efeito tranquilizador e oferecer suporte à comunicação. Na sua circulação por diferentes setores, clínicos ou administrativos, o cão de serviço foi usado como suporte lúdico para a enunciação de queixas e do mal-estar dos trabalhadores. Dirigiam-se a ele na fabulação de monólogos em tom irônico, frequentemente na presença de seus superiores hierárquicos, expressando seu descontentamento frente aos ordenamentos institucionais.

“*Até na véspera do feriado te colocaram pra trabalhar?*” (residente).

“*Você já voltou das férias? Tô vendo! Tá com cara de cansada. Te entendo totalmente*” (médico).

“*Você, que é doutora, bem que podia me dar um atestado para eu ficar uns dias em casa*” (funcionário do arquivo).

“*Deixa eu te contar, eu tô mais cansada que você. Eu vou ficar deitada igual a você*” (técnica de enfermagem).

Outras vezes seus privilégios eram destacados para demarcar um contraponto com a própria situação laboral:

“*Semana que vem eu vou vir de Golden pra receber umas massagens*” (bolsista).

“*Olha só, ela chega de casa no carro do hospital. Nunca andei nesse carro!*” (enfermeira do ambulatório de Pediatria).

“*Ah, você voltou! Bem-vinda de volta! Eu quero um emprego desse, que fica de férias no verão*” (médico da unidade intermediária).

“*Presta atenção nisso: até a cachorra tem crachá e eu ainda não tenho*” (técnica de enfermagem terceirizada do CTI pediátrico).

“*Ela deve ganhar mais que a gente!*” (funcionária de serviços gerais da enfermaria se referindo ao cão de visitação).

Na direção inversa, na presença dela, as queixas também se dirigiam das chefias para seus funcionários:

“*Olha lá, ela está melhor que os funcionários do hospital!* (...) *Esse aí você pode morder, está sem crachá!*” (chefe da manutenção na frente de seus subordinados que circulavam sem portar seu crachá de identificação ao ver o crachá do cão de serviço).

“*Você está parecendo as técnicas de enfermagem daqui: bobeou, deita e já quer dormir!*” (chefe de enfermagem, na presença de suas funcionárias).

Nesse contexto, foi atribuída ao animal a função de porta-voz privilegiado. Através dele, estavam sendo veiculadas, num discurso indireto, algumas verdades ditas na forma de gracejos, que serviam para apontar denúncias e um desgaste físico e emocional comum a muitos trabalhadores em um ambiente regido por tensões, disputas e poder. Seguindo a TAR ^3^, a partir das interações amistosas estabelecidas na presença da cadela de serviço, uma nova oportunidade de expressão de conteúdos indesejáveis estava sendo construída, em uma atmosfera de leveza e suspensão provisória das defesas pessoais. Seria interessante que pudéssemos ficar com o problema, como nos sugere Donna Haraway ^24^, no sentido de que a interação interespécies pode ser potente para revelar o que não vemos ou com que não queremos entrar em contato.

Pode-se entender que o animal, na sua posição a princípio ingênua e espontânea, teria um salvo-conduto por não atuar dirigido pela racionalidade, funcionando, portanto, como um anteparo para possíveis réplicas desrespeitosas ou agressivas contra tais comentários provocadores. O que fosse dito por seu intermédio teria um peso diferente e não seria tomado como uma afronta ou como algo mal-intencionado. Por ter conquistado um lugar de afeto entre os trabalhadores, passou a gozar de um *status* especial que o autorizaria a realizar extravagâncias. Dessa forma, quando passou a participar da cena hospitalar, o animal permitiu que se tornasse visível e dizível o que antes circulava de forma tácita como uma gramática das entrelinhas e dos interditos.

Um enunciado irônico “*faz ouvir uma voz diferente da do ‘locutor’, a voz de um ‘enunciador’ que expressa um ponto de vista insustentável. O ‘locutor’ assume as palavras, mas não o ponto de vista que elas representam*” ^25^ (p. 77). A ironia encerra uma expressão polifônica que possibilita praticar uma negação do sentido literal, às custas da dissimulação do locutor na apropriação do discurso do outro para manifestar uma agressividade cínica ^26^.

Segundo Cattelan: “*Uma ocorrência irônica faria, concomitantemente, serem ouvidas duas vozes, em contraposição, em desacordo: a do locutor que representa uma outra voz (não necessariamente ocorrida, empírica), em relação à qual se mantém em recuo, e a do enunciador que teria produzido um enunciado com que o locutor não se identifica. O locutor simula esta voz, que não é sua, marcada por algum índice de distanciamento, sobre a qual ele imprime um efeito de ridicularização ou de deboche, fazendo ouvir a sua voz, elevando-se à condição de enunciador e se tornando responsável por um ponto de vista*” ^27^ (p. 46).

Dessa forma, “*a ironia é uma aposta, uma tática astuciosa, que põe na boca do outro palavras que não disse, mas que passam por terem sido ditas, enquanto também permite que o locutor negue ter dito o que disse*” ^27^ (p. 51). Nota-se o recurso à ironia, nesse contexto, como uma modalidade de crítica a algo ou alguém, tomando proveito da figura simpática e empática da cadela como um elemento providencial e atenuante para as mensagens ácidas que naquela oportunidade puderam ser enunciadas.

Haraway ^4^ sustenta uma reconfiguração dos modos de viver, das formas de fazer e pensar a partir da abertura de espaço para companhias inesperadas. Ela aciona a categoria espécie companheira para tensionar e recusar o excepcionalismo humano, assumindo parceiros ontologicamente heterogêneos na cocriação de modelos relacionais semiótico-materiais. Quando iniciamos essa pesquisa, estávamos dispostas a, como Haraway propõe, nos enovelar em linhas de conexões inventivas com outros agentes num processo gerativo de “devir-com” na experimentação de novas cooperações. Instigadas por suas ideias, acompanhamos e cooperamos na tessitura dessa trama dinâmica interespécie. Latour ^3^ e Haraway ^4^ se alinham na perspectiva interacional e materialista de como os coletivos são “construídos-com” outros de maneira recíproca, e não só por humanos.

O trecho abaixo captura uma reordenação propiciada por essa parceria humano-animal:

“*A senhora não me leve a mal, mas toda vez que eu te vejo, te associo ao cachorro. Quando é que ela vai vir aqui de novo?*” (funcionária do apoio administrativo em diálogo com a pesquisadora, que é profissional de saúde do hospital).

O animal tornou-se para os trabalhadores o protagonista da cena, centro dos interesses e atenção. No curso das visitas houve uma reformulação da identidade da profissional de saúde coordenadora do PAAA, que passou a ser conhecida e reconhecida na comunidade hospitalar como a “*moça do cachorro*”. Além dela, toda a equipe responsável pela organização da atividade passou a gozar de um tratamento privilegiado dentro do hospital, não apenas nos processos referentes à gestão das visitas. Estarmos associadas ao cão de serviço nos outorgou um status especial dentro da comunidade de trabalhadores, facilitando contatos e acessos. Diversas vezes e em diferentes contextos ouvimos: “*podem contar comigo para o que precisarem, estou sempre à disposição*”.

Outro deslocamento foi produzido com a presença regular da cadela de serviço. Observou-se o surgimento espontâneo de um movimento contrário de visitação, que tornava a cadela o indivíduo a ser buscado por profissionais de saúde, familiares e crianças, levantando a questão de quem estava visitando quem naquele contexto. A visita do animal passou a ser também tema de conversas e ponto de contato entre trabalhadores que possivelmente não se aproximariam de outra forma.

“*Quando ela chega, a gente vai ver ela, vem outras pessoas que eu nunca vi, que não conheço, e acaba se interagindo ali de conversar. Parece que quando ela chega dá uma quebrada na rotina, todo mundo se fala, há uma interação com todo mundo, pessoas de setores diferentes. Que a gente passa a se falar depois*” (funcionária administrativa do setor de apoio do hospital).

O animal atua como mediador de encontros que de outra forma seriam improváveis, promovendo pontes entre atores e facilitando uma ambiência relacional mais saudável e leve no espaço hospitalar. Como excelentes “lubrificantes sociais”, os cães são capazes de conectar as pessoas e aumentar a autoestima delas ^22^. Esse efeito alcança uma dimensão política, sobretudo quando consideramos os trabalhadores dos bastidores do cuidado como aqueles que compõem uma rede de suporte técnico-administrativo para a assistência ofertada na ponta aos usuários. Na cadeia de serviços dentro do hospital, o trabalho que desenvolvem habitualmente é pouco reconhecido e valorizado. Ao compartilhar seu tempo e presença junto a estes, o animal, que não reconhece hierarquias sociais e tem suas preferências para além de qualquer status na instituição, opera um reposicionamento da cadeia de valores, colapsando momentaneamente a estrutura vertical que localiza os indivíduos segundo seu poder ou nível acadêmico. Outros referenciais simbólicos e relacionais passam a ser acionados quando a cadela de serviço entra em cena, rearticulando as tramas cotidianas como um agente político que transforma as dinâmicas interpessoais e interespécies.

## Considerações finais

A relação humano-animal no espaço de cuidado hospitalar foi investigada a partir da observação densa e extensa de um programa de apoio assistido por animais na visitação regular de uma única cadela de serviço a crianças e adolescentes, internados em um hospital público terciário especializado na saúde materno-infantil, no Rio de Janeiro.

Todos os efeitos observados e discutidos neste artigo parecem depreender, em última análise, do fenômeno que podemos descrever como um relaxamento das regras sociais que orientam as interações no hospital.

Um cão de serviço no hospital é mais que um ser individual. Ele assume um corpo coletivo de representação simbólica de todos os animais com os quais se tem um vínculo afetivo significativo. Sua presença aciona e opera mecanismos distintos daqueles vigentes nas relações inter-humanas.

Valorizar abordagens que ampliam os horizontes da sociabilidade na consideração e incorporação de outros agentes do cuidado na cena hospitalar, como o cão de visitação, contribui para a inovação e qualificação das tecnologias em saúde, destacando a dimensão micropolítica das emoções na reorganização das relações interpessoais e interespécies.

Cabe reconhecer o valor do cuidado sustentado por lógicas não orientadas pela racionalidade e que promovem o encontro espontâneo e genuíno a partir de um movimento que desconsidera poder e hierarquia social, promovendo uma ruptura paradigmática na ordenação das interações dentro do hospital. A atuação da cadela de serviço, funcionando quase como um corpo público, reorganiza e reconfigura a rede de relações estabelecendo novos pontos de contato, a partir de sua participação regular no espaço. Nos diferentes agenciamentos que produz, e nas mensagens articuladas ou silenciosas que por seu intermédio são veiculadas, notabiliza-se o papel político edificado na economia dos afetos no espaço de cuidado.

## Data Availability

Os dados de pesquisa não estão disponíveis.
